# The lncRNA *Firre* anchors the inactive X chromosome to the nucleolus by binding CTCF and maintains H3K27me3 methylation

**DOI:** 10.1186/s13059-015-0618-0

**Published:** 2015-03-12

**Authors:** Fan Yang, Xinxian Deng, Wenxiu Ma, Joel B Berletch, Natalia Rabaia, Gengze Wei, James M Moore, Galina N Filippova, Jun Xu, Yajuan Liu, William S Noble, Jay Shendure, Christine M Disteche

**Affiliations:** Department of Pathology, University of Washington, Seattle, Washington 98195 USA; Department of Genome Sciences, University of Washington, Seattle, Washington 98195 USA; Division of Human Biology, Fred Hutchinson Cancer Research Center, Seattle, Washington 98109 USA; Department of Integrative Physiology and Neuroscience, Washington State University, Pullman, Washington 99164 USA; Department of Computer Science and Engineering, University of Washington, Seattle, Washington 98155 USA; Department of Medicine, University of Washington, Seattle, Washington 98195 USA

## Abstract

**Background:**

In mammals, X chromosome genes are present in one copy in males and two in females. To balance the dosage of X-linked gene expression between the sexes, one of the X chromosomes in females is silenced. X inactivation is initiated by upregulation of the lncRNA (long non-coding RNA) *Xist* and recruitment of specific chromatin modifiers. The inactivated X chromosome becomes heterochromatic and visits a specific nuclear compartment adjacent to the nucleolus.

**Results:**

Here, we show a novel role for the lncRNA *Firre* in anchoring the inactive mouse X chromosome and preserving one of its main epigenetic features, H3K27me3. Similar to *Dxz4*, *Firre* is X-linked and expressed from a macrosatellite repeat locus associated with a cluster of CTCF and cohesin binding sites, and is preferentially located adjacent to the nucleolus. CTCF binding present initially in both male and female mouse embryonic stem cells is lost from the active X during development. Knockdown of *Firre* disrupts perinucleolar targeting and H3K27me3 levels in mouse fibroblasts, demonstrating a role in maintenance of an important epigenetic feature of the inactive X chromosome. No X-linked gene reactivation is seen after *Firre* knockdown; however, a compensatory increase in the expression of chromatin modifier genes implicated in X silencing is observed. Further experiments in female embryonic stem cells suggest that *Firre* does not play a role in X inactivation onset.

**Conclusions:**

The X-linked lncRNA *Firre* helps to position the inactive X chromosome near the nucleolus and to preserve one of its main epigenetic features.

**Electronic supplementary material:**

The online version of this article (doi:10.1186/s13059-015-0618-0) contains supplementary material, which is available to authorized users.

## Background

Mammalian X chromosome inactivation (XCI) results in random silencing of one of the two X chromosomes in females in order to balance the dosage of X-linked gene expression between the sexes [1]. XCI is initiated by upregulation of the long non-coding RNA (lncRNA) *Xist* triggered by the loss of pluripotency factors during early development [2,3]. *Xist* RNA coats the inactive X chromosome (Xi) *in cis* and recruits specific chromatin modifiers for silencing [4,5]. Notably, the polycomb repressive complex 2 (PRC2) methylates histone H3 at lysine 27 (H3K27me3), leading to chromatin compaction [6]. Other chromatin modifications that accumulate on the inactive X are H3K9me2-3, H2AK119ub, and H4K20me1. Later, additional epigenetic changes such as DNA methylation at CpG islands, deposition of macroH2A, and late replication lock in silencing to ensure stability and faithful transmission of the inactive state to daughter cells [7,8]. The primary silencing step, that is, *Xist* expression, was initially thought to be dispensable for the maintenance of XCI [9], but subsequent studies show that *Xist*, in synergy with epigenetic modifications on the Xi, is needed for long-term persistence of silencing [10]. Interestingly, loss of *Xist* or of anyone of the histone modifications, for example H3K27me3 or macroH2A, is not sufficient to induce rapid X-linked gene reactivation in somatic cells, confirming that stable long-term silencing relies on several layers of epigenetic changes [11-15].

Although most genes on the Xi are silenced, some genes escape XCI and remain expressed within the heterochromatic context [16]. In mice, only about 3% to 6% of mouse X-linked genes consistently escape XCI based on RNA-sequencing analyses using single nucleotide polymorphisms (SNPs) to distinguish expression from each allele [17-20]. Escape from XCI results in significant sexual dimorphisms in levels of gene expression, suggesting that escape genes may be important for female-specific functions, including XCI. One of the genes found to escape XCI in human and mouse is the lncRNA *FIRRE*/*Firre* (previously named *6720401G13Rik* in mouse), which represents a macrosatellite repeat located a great distance away from the XIC (X inactivation center) [17,21]. *Firre* has previously been shown to bind hnRNPU and serve as a platform for trans-chromosomal associations involved in the regulation of pluripotency pathways in male embryonic stem (ES) cells [21], but its role in relation to XCI has not been investigated.

The position of the X chromosomes within the nucleus changes during initiation and maintenance of XCI [22]. Prior to XCI the two active X chromosomes adopt random positions in the nucleus, followed by pairing to ensure correct sensing and counting [23,24]. Once chosen, the Xi forms the heterochromatic Barr body that occupies a discrete and condensed compartment from which active epigenetic marks are excluded [25,26]. Furthermore, the Xi frequently visits the perinucleolar region during S phase, probably for heterochromatin replication and maintenance [27]. Factors important in such positioning are still elusive. One candidate is the 11-zinc finger protein CTCF (CCCTC binding factor). CTCF has been implicated in diverse functions throughout the genome, including control of transcription, promoter/enhancer interactions, chromatin insulation, and nuclear organization [28-30]. This functional diversity depends on which of the 11 CTCF zinc fingers binds to chromatin [31], and on factors that form complexes with CTCF [32]. In concert with nucleophosmin, cohesin, and/or A-type lamins, CTCF tethers chromatin to specific nuclear compartments for gene regulation [33-36]. Interestingly, immunofluorescence analyses show a focal accumulation of CTCF inside the Barr body [26,37], which overlaps with the lncRNA *DXZ4* locus known to specifically binds CTCF on the Xi [38,39]. Potential interactions between *DXZ4* and *FIRRE* have been reported and Hi-C studies further show that the Xi condenses in two major domains separated by *DXZ4* in human cells, suggesting a role in the Xi structure [39,40].

To investigate the role of *Firre* in mouse XCI we performed allele-specific analyses to demonstrate that this locus specifically binds CTCF and cohesin on the Xi. The *Firre* locus on the Xi but not the Xa (active X) was found to be located adjacent to the nucleolus. Knockdown of *Firre* RNA in mouse fibroblasts disrupted this perinucleolar location and caused a decrease in H3K27me3 enrichment especially on the X chromosome, whereas knockdown in female ES cells had no effects on XCI onset as determined by *Xist* RNA accumulation nor on *G6pdx* gene silencing. These studies reveal a novel role for a lncRNA located away from the XIC in positioning and in maintenance of a specific epigenetic feature of the silenced X chromosome in somatic cells.

## Results

### *Firre* binds CTCF and cohesin specifically on the inactive X chromosome

**Figure 1:**
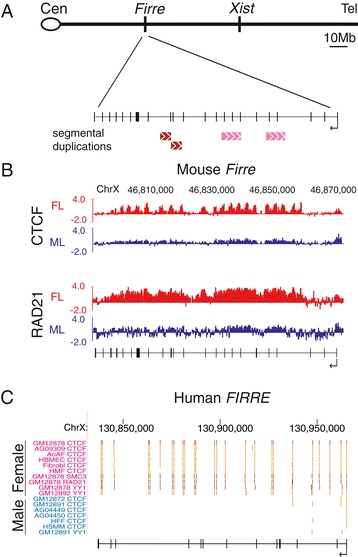
**The** ***Firre/FIRRE*** **loci bind CTCF and RAD21 in mouse and human females. (A)** Schematic of the mouse X chromosome showing the location of *Firre*, and *Xist*. Blow-up shows the lncRNA *Firre* (transcription direction marked by an arrow) with the location of segmental duplications of tandem repeats (same color indicates paired duplicated regions). Cen, centromere; Tel, telomere. **(B)** CTCF and RAD21 are bound to *Firre* in female liver (FL) but not in male liver (ML). ChIP-chip data are shown as log_2_ ChIP/input. Genomic coordinates are shown at top. **(C)** CTCF, cohesin (SMC3 and RAD21), and YY1 are bound to *FIRRE* in female (red) but not in male human B-lymphocytes (blue). Peak center tracks (darker color indicates peak strength) from the human ENCODE project [45]. Genomic coordinates are shown at top.

Comparisons of CTCF occupancy profiles between male and female mouse tissues by ChIP-chip identified a striking female-specific CTCF-binding cluster located at the lncRNA *Firre* locus several Mb away from the XIC (Figure 1). The *Firre* locus is approximately 80 kb in length and contains two subregions, each tandem-duplicated and arranged head-to-tail, thus meeting the definition of a macrosatellite repeat (Figure 1A). One set of duplicons (3.4 kb) has 94% sequence identity and the other (6.2 kb), 92% identity. The macrosatellite repeat is GC-rich and enriched in simple repeats, but is remarkably devoid of LINE repeats. RAD21, a component of the cohesin complex, was also bound to *Firre* only in female mouse tissues (Figure 1B) as expected from the known frequent co-localization of CTCF and cohesin [41-44]. The homologous *FIRRE* locus in human is also enriched in CTCF, as well as in two cohesin components (RAD21, SMC3) and YY1 specifically in female but not male cells based on occupancy profiles from ENCODE (Figure 1C) [45].

**Figure 2:**
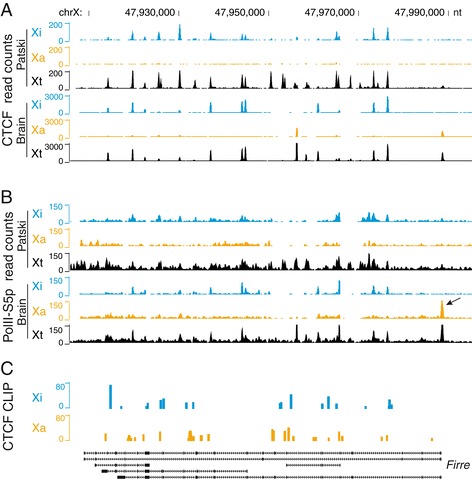
**CTCF and RNA polymerase II are present at the** ***Firre*** **locus on the Xi allele and CTCF interacts with the lncRNA.** Allele-specific CTCF **(A)** and PolII-S5p **(B)** profiles were established based on SNP analyses of ChIP-seq results in Patski cells and in F1 mouse brain, both derived from crosses between C57BL/6 J and *M. spretus*. In Patski cells the Xi is from BL6 while in the brain, the Xi is from *spretus* X. Allele-specific sequence read counts for the Xi (blue) and the Xa (orange), and total mapped read counts (Xt) are shown in 100 bp windows. A few peaks visible in the Xt track could not be assigned to an allele due to a lack of informative SNPs. PolII-S5p peaks are present in multiple regions of *Firre* on the Xi, indicating alternative transcript start sites. The arrow points to a PolII-S5p peak present only on the Xa at the very 5’end of the *Firre* transcript in brain. Genomic coordinates are shown at the top and the position of alternative *Firre* transcripts, at the bottom (from UCSC). **(C)** CTCF-RNA interactions obtained by CLIP-seq based on re-analysis of published data [49]. Allele-specific profiles for the Xi (blue) and Xa (orange) show differential patterns of interactions between CTCF and the lncRNA transcribed from each allele.

To examine CTCF occupancy on the mouse Xa and Xi separately, allele-specific ChIP-seq was done using *in vitro* and *in vivo* F1 mouse systems in which XCI is completely skewed and alleles differ by frequent SNPs (1 per 50 to 100 bp) [17,46,47]. Allele-specific CTCF occupancy profiles generated using Patski cells (Xi from C57BL/6J) or adult brain (Xi from *Mus spretus*), showed that CTCF binding was specific to the Xi allele of the *Firre* locus (Figure 2A). CTCF motif analysis using FIMO (Find Individual Motif Occurrences) [48], based on a previous study that identified three types of motifs: core (C), upstream (U), and downstream (D) [31], showed that most CTCF motifs within the *Firre* locus were of the common C-type (68/70 on the BL6 allele and 65/67 on the *spretus* allele), with only two motifs of the rarer D-type (in the first and third introns). Allele-specific occupancy profiles of RNA polymerase II phosphorylated at serine 5 (PolII-S5p), which is associated with active transcription, showed similarities to the CTCF occupancy profiles, in agreement with the known role of CTCF in transcription (Figure 2B). Note that there were striking differences in the PolII-S5p profiles between the Xi and Xa (see below). Next, we tested whether CTCF interacts with *Firre* RNA itself by re-analysis of published data on allele-specific CTCF-RNA binding in mouse ES cells after 3 days of differentiation [49]. CTCF was found to bind to *Firre* RNA transcribed from either the Xa or the Xi, with some differences between alleles (Figure 2C). Thus, CTCF interacts with the *Firre* genomic locus on the Xi as well as with the lncRNA itself.

A second cluster of CTCF binding sites on the Xi was observed at *Dxz4*, another macrosatellite repeat associated with a lncRNA, *4933407K13Rik*, confirming previous observations [50]. The CTCF binding sites within the mouse *Firre* and *Dxz4* loci were found to be highly conserved in human (>90% identity), suggesting that the CTCF-based functions of these regions are conserved between mammalian species.

### Sex-specific changes in CTCF binding and expression of *Firre* and *Dxz4* during development

**Figure 3:**
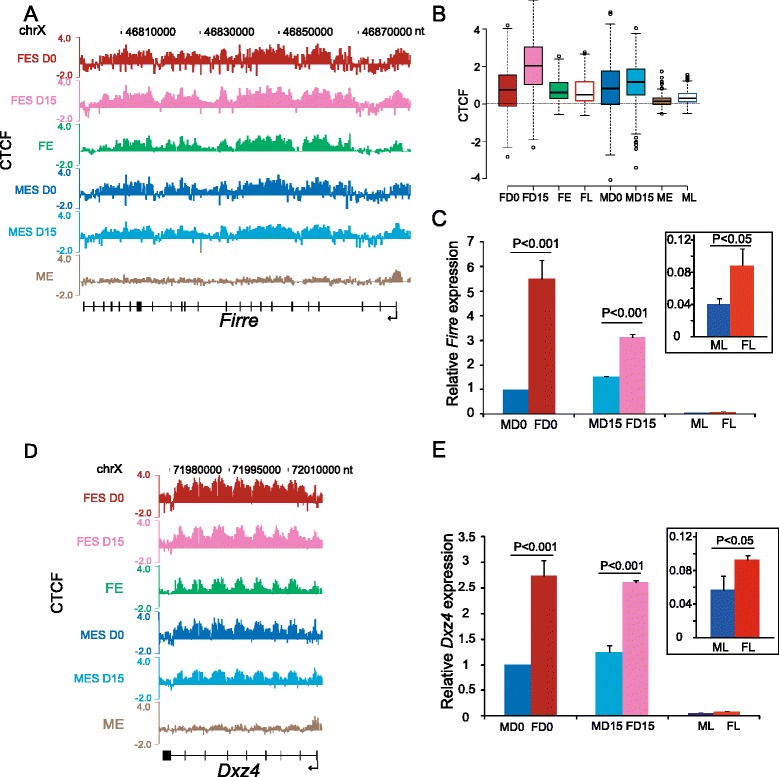
**CTCF binding and expression change during male and female development. (A)** CTCF occupancy at *Firre* in mouse female ES cells PGK12.1 before (FES D0) and after 15-day differentiation (FES D15) and in female 12.5 dpc embryos (FE) is compared to occupancy in male mouse ES cells WD44 before (MES D0) and after 15-day differentiation (MES D15) and in male 12.5 dpc embryos (ME). ChIP-chip data are shown as log_2_ ChIP/input. **(B)** Box plots of CTCF occupancy (log_2_ ChIP/input) at *Firre* in male (MD0, MD15) and female (FD0, FD15) ES cells, 12.5 dpc embryos (ME, FE), and adult livers (ML, FL). Same data as in (A) and Figure 1B. **(C)** *Firre* expression level is significantly higher in female than in male ES cells (undifferentiated, D0, differentiated, D15) and liver (box insert). Note that expression is much higher in ES cells than in liver. Expression in male ES cells (D0) is set as 1. Average expression levels were measured by qRT-PCR of biological replicates and normalized to 18S RNA. *P* values were determined by one sample t-test. Error bars, s.e.m. **(D, E)** Same analysis as in (A, C) for *Dxz4*.

To follow CTCF binding at the *Firre* locus before and after the onset of XCI, ChIP-chip was performed in female (PGK12.1) and male (WD44) mouse ES cells before (day 0) and after differentiation (day 15), as well as in mouse embryos (12.5 dpc) and adult tissue (liver). Surprisingly, the *Firre* locus was bound by CTCF in both female and male ES cells at day 0, indicating binding to the Xa in undifferentiated ES cells (Figure 3A). At day 15 after differentiation median CTCF occupancy at the locus increased in female but not in male ES cells (Figure 3B). At later stages of male development (12.5 dpc embryos and adult liver) CTCF binding decreased, whereas it persisted in the corresponding female tissues, consistent with loss of binding on the Xa and retention on the Xi (Figures 1B, 2A, 3A,B). Accordingly, median CTCF occupancy at the locus was significantly decreased in male embryos (38% decrease) and liver (30% decrease) compared to ES cells (*P* <2e-16, Wilcoxon Rank-Sum test; Figure 3B), while there was no significant change between the corresponding stages in females (*P* = 0.86 and 0.24, respectively) (Figure 3B). Similar changes were observed at the *Dxz4* locus (Figure 3D). Thus, CTCF binding at the *Firre* and *Dxz4* loci is specifically maintained only on the Xi.

We next examined *Firre* expression during development. By quantitative RT-PCR (qRT-PCR) the level of *Firre* RNA was much higher in ES cells than in adult liver (Figure 3C), consistent with expression levels we previously reported in mRNA-seq datasets (14 RPKM (reads per kilobase of exon per million mapped reads) in PGK12.1 and 0.9 RPKM in mouse liver) [47]. Note that the fold change detected by qRT-PCR analysis was greater than that detected by RNA-seq, probably because the latter only detects mature RNA with polyA tails, while the RT-PCR amplicon may target alternative and/or short transcripts from the macrosatellite repeat locus (Additional file 1: Figure S1). As expected given that *Firre* escapes XCI [17,21], expression was significantly higher in females not only in undifferentiated ES cells prior to XCI but also in differentiated ES cells and in liver, compared to the corresponding male tissues (Figure 3C). *Firre* expression from both the Xi and the Xa was supported by PolII-S5p occupancy on both alleles: 10 to 12 peaks of high PolII-S5p occupancy were dispersed throughout the locus on the Xi, while a single peak was located at 5′ end on the Xa (Figure 2B). Based on these striking differences we conclude that *Firre* transcripts differ between the Xi and Xa, with multiple alternative start sites on the Xi, possibly generating small transcripts. The lncRNA *4933407K13Rik* transcribed at the *Dxz4* locus was also more highly expressed in female than male cells and liver (Figure 3E). Interestingly, different size transcripts are also generated from each *DXZ4* allele in human cells, with short transcripts specifically originating from the Xi [51].

We performed RNA-FISH for *Firre* in Patski cells to determine the lncRNA location. Surprisingly, no *Firre* RNA signal was detected from the Xi marked by the *Xist* RNA cloud and only one bright signal presumably from the Xa was observed (Additional file 2: Figure S2A). This was confirmed using RNA-FISH in female MEFs (mouse embryonic fibroblasts) and in primary neuronal cells derived from the mouse hippocampus (Additional file 3: Figure S3). The failure to detect a *Firre* RNA signal on the Xi even though RNA-seq and PolII occupancy profiles clearly indicate transcription from the Xi could be due to low Xi-expression that represents only 16% of Xa-expression based on allelic RNA-seq (Additional file 4: Table S1), and/or to low sensitivity of RNA-FISH for the detection of short alternative transcripts on the Xi. Likewise, a previous study reported that RNA-FISH failed to detect a signal on the Xi in mouse lung fibroblasts unless *Firre* was ectopically overexpressed many folds [21].

### *Firre* and *Dxz4* alleles on the inactive X chromosome associate with the nucleolus

**Figure 4:**
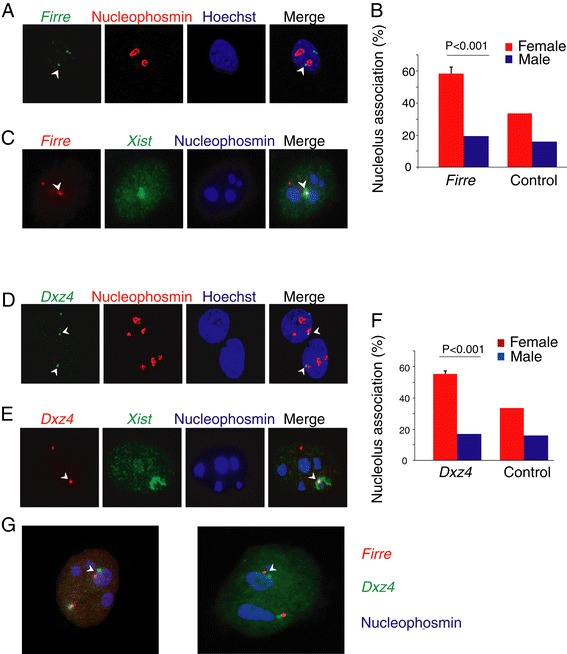
***Firre*** **and** ***Dxz4*** **on the Xi are associated with the nucleolus in female nuclei. (A)** Examples of nuclei from Patski cells after DNA-FISH to detect *Firre* (green signals), immunostaining to detect nucleophosmin (red) on the surface of the nucleolus, and counterstaining with Hoechst 33342 (blue). *Firre* association with the nucleolus is marked by an arrowhead. One deconvoluted Z section is shown. **(B)** Frequency of association (in % of nuclei) between the nucleolus and *Firre* or a control BAC clone (RP23-112D6) in female Patski cells (red) and male ear fibroblasts (blue). *P* values were determined by one sample t-test. Error bars, s.e.m. **(C)** Examples of nuclei from Patski cells after DNA-FISH to detect *Firre* (red), RNA-FISH to detect *Xist* (green) and mark the Xi, and immunostaining to detect nucleophosmin (blue). One deconvoluted Z section is shown. **(D-F)** Same analysis as in A-C for *Dxz4* (see also Additional file 5: Figure S4D). **(G)** *Firre* and *Dxz4* on the Xi are separately associated with the nucleolus in female nuclei. Example of nuclei from Patski cells after DNA-FISH to detect *Firre* (red), *Dxz4* (green), and immunostaining to detect nucleophosmin (blue) on the surface of the nucleolus (see also Additional file 5: Figure S4A).

The location of the *Firre* and *Dxz4* loci within the nucleus was determined by DNA-FISH using labeled BAC clones (RP23-338M16 for *Firre* and RP23-299L1 for *Dxz4*) in combination with immunofluorescence with an anti-nucleophosmin antibody. At least 100 nuclei were scored for each experiment to evaluate the association between the loci and the nucleolus, as defined by the adjacent location of one FISH signal to the nucleolus surface (Additional file 5: Figure S4). *Firre* association to the nucleolus was much more frequent in female compared to male cells (Figure 4A; Additional file 5: Figure S4A-C). Indeed, we observed association in 58% of female fibroblasts (Patski), but only in 17% of male ear fibroblasts (Figure 4B). Since female cells have two X chromosomes the predicted percentage of nuclei with background nucleolar association for at least one signal was estimated to be 34% based on observations in male nuclei. Accordingly, a control BAC clone (RP23-112D6) for a region not enriched in CTCF binding showed background levels of nucleolar association in 34% and 16% of female and male fibroblasts, respectively (Figure 4B). The increased association of the *Firre* locus with the nucleolus surface in female versus male cells was highly significant (*P* = 0.0008 by one-sample t-test). To identify the *Firre* allele that associated with the nucleolus surface the Xi domain was marked by RNA-FISH using an *Xist* probe labeled a different color. A clear overlap between the *Xist* RNA cloud and the *Firre* DNA signal located adjacent to the nucleolus identified the Xi allele as the one targeted to the perinucleolar region (Figure 4C). Similar results were obtained for *Dxz4* (Figure 4D-F; Additional file 5: Figure S4D,E). However, DNA-FISH signals obtained using different color probes for the two loci did not overlap, suggesting that the *Firre* and *Dxz4* loci did not aggregate within a single complex (Figure 4G, Additional file 5: Figure S4A). Based on these results, we conclude that there is independent association between the two CTCF-binding clusters on the Xi and the nucleolus surface.

### Knockdown of *Firre* RNA decreases perinucleolar localization of the Xi in fibroblasts

**Figure 5:**
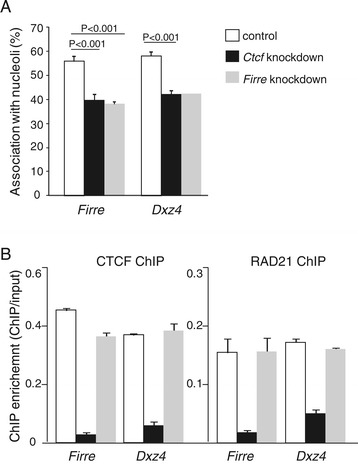
**Knockdown of** ***Firre*** **or** ***Ctcf*** **decreases association of** ***Firre*** **and** ***Dxz4*** **to the nucleolus. (A)** Association frequency (in % of nuclei) between *Firre* or *Dxz4* and the nucleolus as determined by DNA-FISH and immunostaining is significantly reduced after either *Firre* or *Ctcf* knockdown in Patski cells. At least 100 nuclei were scored in each experiment. *P* values were determined by two-tail unpaired student t-test. Error bars represent s.e.m. **(B)** Occupancy by CTCF and RAD21 at *Firre* and *Dxz4* before and after knockdowns of *Firre* or *Ctcf*. ChIP enrichment is shown as the ratio between ChIP and input fractions as measured by qRT-PCR. Error bars represent s.e.m.

We next examined the effects of *Firre* RNA depletion. Knockdown of *Firre* using a double siRNA (small interfering RNA) treatment in Patski cells achieved a >70% reduction in levels of the lncRNA and resulted in a significantly lower frequency of association between *Firre* and the nucleolus (from 56% to 40%; *P* = 0.0002 by two-tail unpaired student t-test; Figure 5A). The frequency of *Dxz4* association to the nucleolus was also reduced (from 58% to 42%, *P* = 0.005 by one-sample t-test), suggesting that the perinucleolar location of the whole Xi was disrupted (Figure 5A). Since no changes in CTCF and RAD21 occupancy were apparent at the *Firre* (or *Dxz4*) locus after knockdown, *Firre* lncRNA levels apparently influence perinucleolar targeting of the Xi independent of CTCF and RAD21 binding at the locus (Figure 5B). We also performed *Ctcf* knockdown and verified depletion by qRT-PCR and western blots (Additional file 6: Figure S5A). Knockdown of *Ctcf* also significantly reduced perinucleolar targeting of both *Firre* (from 56% to 39%, *P* = 0.0009) and *Dxz4* (from 58% to 42%, *P* = 0.0004), implying that CTCF was necessary for targeting of the Xi (Figure 5A). This effect could be indirect since *Ctcf* knockdown caused a significant reduction in expression of *Firre* (but not of *4933407K13Rik*, data not shown). Furthermore, *Xist* expression was also decreased (see below), consistent with a previous report in differentiated mouse ES cells [52], and possibly causing a loss in nucleolus association of the Xi [27]. In contrast, no *Xist* reduction was observed after *Firre* knockdown, suggesting that *Firre* acts independently from *Xist*. There was a remarkable loss of RAD21 at *Firre* (and at *Dxz4*) after *Ctcf* knockdown, indicating that cohesin binding at these loci is dependent on CTCF (Figure 5B). Taken together, our results suggest that the lncRNA *Firre* and CTCF occupancy at its genomic locus may help anchoring the Xi to the nucleolus.

### *Firre* and *Ctcf* knockdowns in fibroblasts cause a loss of H3K27me3 on the Xi

**Figure 6:**
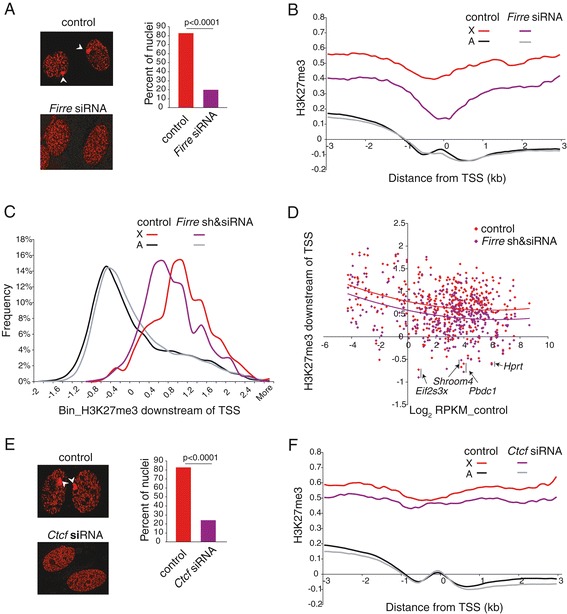
**Knockdown of** ***Firre*** **or** ***Ctcf*** **affects H3K27me3 enrichment of the Xi. (A)** Examples of interphase nuclei after H3K27me3 immunostaining (left panel) and graph of percent of nuclei with an H3K27me3 intense staining spot (right panel) in control and *Firre* knockdown Patski cells. The H3K27me3 spot over the Xi (marked by arrowheads) is no longer visible after knockdown. *P* value was determined by Chi-square tests. **(B)** H3K27me3 enrichment is reduced at the 5’ end of X-linked genes but not at autosomal genes after *Firre* knockdown using siRNAs in Patski cells. Metagene analysis shows average H3K27me3 enrichment 3 kb upstream and downstream of the transcription start site (TSS) for 647 X-linked genes (X) versus 16,141 autosomal genes (A). **(C)** The distribution of average H3K27me3 enrichment at 1 to 2 kb downstream of the TSS is clearly shifted left to lower values for X-linked genes but not for autosomal genes after *Firre* knockdown. **(D)** Scatter plot of H3K27me3 enrichment at 1 to 2 kb downstream of the TSS shows a shift to lower values for most X-linked genes after *Firre* knockdown (purple) compared to control (red), as indicated by trend lines. Three escape genes, *Eif2s3x*, *Shroom4*, and *Pbdc1* that show no H3K27me3 enrichment are labeled. *Hprt*, a gene subject to XCI, shows no H3K27me3 enrichment because it is deleted in Patski cells. **(E, F)** Same analysis as in (A, B) but for *Ctcf* knockdown. There is a lesser decrease in H3K27me3 levels on the X than in *Firre* knockdown.

To determine whether *Firre* influences epigenetic features of the Xi H3K27me3 enrichment was compared before and after RNA knockdown. Independent *Firre* RNA knockdowns were done using either a double siRNA treatment or a combination of shRNA and siRNA in Patski cells. Immunostaining of H3K27me3 in interphase nuclei showed a significant reduction by 76% in the number of nuclei with an intense spot corresponding to the Xi after *Firre* knockdown, consistent with a substantial loss of H3K27me3 normally detected on the Xi (Figure 6A). RNA-FISH showed no significant changes in the frequency or shape of *Xist* RNA clouds visible in 89% of *Firre* knockdown cells compared to 87% of control cells (Additional file 2: Figure S2B). This, together with no change in *Xist* RNA levels upon *Firre* knockdown, strongly suggests that *Firre* regulates Xi nucleolus association and H3K27me3 enrichment independent of *Xist*. The loss of H3K27me3 on the Xi was further demonstrated by metagene analyses based on ChIP-chip for H3K27me3 after two independent *Firre* knockdowns, either transient (siRNA) or stable (shRNA and siRNA) (Figure 6B-D; Additional file 6: Figure S5B). As expected based on the known high levels of H3K27me3 on the Xi, average enrichment between +1 kb and +2 kb of the TSS was higher for X-linked genes than for autosomal genes. Importantly, upon *Firre* knockdown, a clear shift to lower H3K27me3 values was specifically observed for X-linked genes whose average enrichment significantly decreased by 15% to 17% (*P* = 1e-10, Kolmogorov–Smirnov test) (Figure 6C). Comparisons between X-linked genes showed a greater decrease in H3K27me3 for genes subject to XCI, while escape genes [17] had low H3K27me3 levels to begin with, and little to no change after knockdown (Figure 6D; Additional file 7: Table S2). Note that, despite being subject to XCI, *Hprt* had no apparent H3K27me3 enrichment, due to its deletion from the Xi in Patski cells (Figure 6D) [46].

After *Ctcf* knockdown a lesser effect on the percentage of nuclei with an intense spot of H3K27me3 (71% decrease) and on the average X-specific enrichment measured by metagene analysis (5% decrease) were observed (Figure 6E, F). We conclude that *Firre* and to a lesser extent CTCF are important for maintenance of H3K27me3 on the Xi.

### Gene expression analyses after *Firre* knockdown in fibroblasts

**Figure 7:**
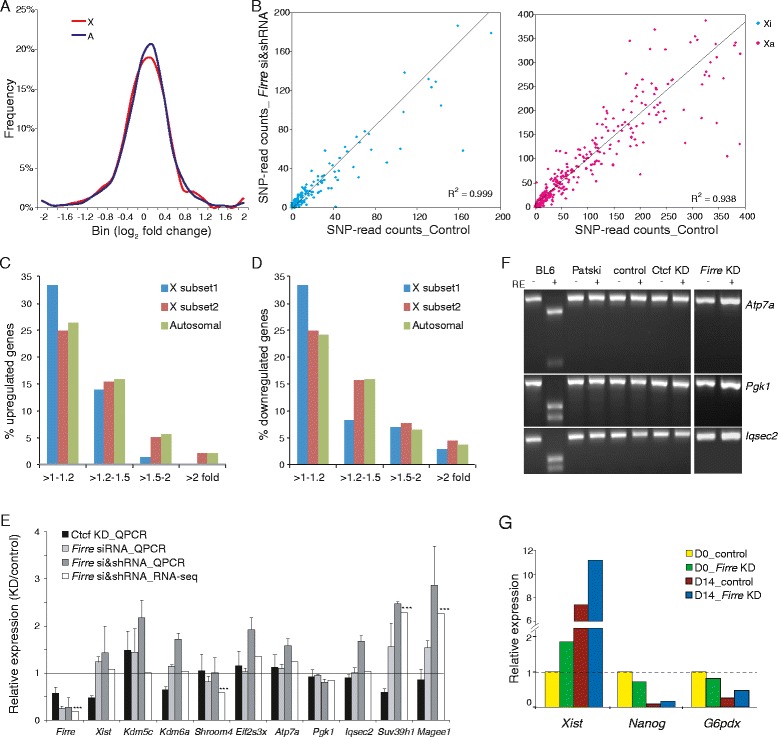
**Effects of** ***Firre*** **or** ***Ctcf*** **knockdown on gene expression. (A)** Similar distributions of expression fold changes for 342 X-linked (X) and 11,182 autosomal genes (A) with ≥1 RPKM in Patski cells between control and stable *Firre* knockdown. **(B)** Scatter plots of SNP-read counts from 434 alleles on the Xi (left panel) or on the Xa (right panel) between control and *Firre* stable knockdown in Patski cells. Only X-linked alleles with at least one SNP-read count in control or knockdown are included. Trend lines and R2 are shown. **(C, D)** Percent of genes upregulated **(C)** or downregulated **(D)** after *Firre* knockdown in Patski cells. X-linked genes are grouped as ‘subset 1’ representing those with ≥5 SRPM (SNP reads per 10 M mapped reads) from the Xi, and ‘subset 2’ representing those with <5 SRPM. **(E)** Expression changes for *Firre*, *Xist*, four escape genes (*Kdm5c*, *Kdm6a*, *Shroom4*, and *Eif2s3x*), and five genes subject to XCI (*Atp7a*, *Pgk1*, *Iqsec2*, *Magee1*, and *Suv39h1*) after *Ctcf* or *Firre* KD (siRNA or si&shRNA treatment). Expression ratios between KD and control measured by qRT-PCR (normalized to *β-actin*) or RNA-seq. Error bars, s.e.m. Genes showing significant expression changes by RNA-seq between *Firre* KD and control cells are indicated (***P <0.001 from Cuffdiff2 analysis). **(F)** No reactivation of *Atp7a*, *Pgk1*, and *Iqsec2* was observed after *Ctcf* or *Firre* KD in Patski cells. Gel images of RT-PCR products without (-) and with (+) restriction enzyme (RE) digestion to specifically cleave BL6 alleles in BL6 cells, untreated Patski cells, and Patski cells treated with control siRNA, *Ctcf* siRNA or *Firre* siRNA are shown. **(G)** Expression changes for *Xist*, *Nanog*, and *G6pdx* in female mouse ES cells PGK12.1 during 14-day EB (embryoid body) differentiation. Expression measured by qRT-PCR normalized by *18S*, and set to 1 in control cells at D0.

RNA-seq was initially used to compare gene expression in Patski cells between control and *Firre* knockdown obtained using a combination of shRNA and siRNA (see Methods for details; Additional file 4: Table S1). For both expressed (≥1 RPKM) X-linked genes (342) and autosomal genes (11,182) the median fold changes were about 1.0 (0 in log_2_ scale; Figure 7A), indicating no overall change in gene expression after knockdown. However, subsets of genes representing 33 (10%) X-linked and 1,218 (11%) autosomal genes were significantly downregulated, while 17 (5%) X-linked and 811 (7%) autosomal genes were significantly upregulated (>1.25-fold expression change and *P* <0.05 from Cuffdiff2 analysis; Additional file 4: Table S1). Similar trends were observed when using 1.5- or 2.0-fold cutoffs. Upregulated genes (but not downregulated genes) were enriched in distinct functional categories such as nucleus, DNA-binding, chromosomal proteins, and chromatin regulating proteins, based on analysis by GO DAVID [53] (Additional file 8: Table S3). Interestingly, a subset of these upregulated genes encode protein components of the PRC1 complex (*Bmi1*, *Cbx6*, and *Rnf2*) and proteins implicated in heterochromatin regulation (*Cbx1*, *Suv39h1*) or in maintenance of DNA methylation (*Dnmt1*). These proteins are known to mediate the deposition of X silencing marks such as H3K9me2-3, H2AK119ub, H2b, and DNA methylation [13] (Additional file 9: Table S4). In contrast, *Suz12*, a gene that encodes a core subunit of the PRC2 complex that controls H3K27me3, had a 30% decrease in expression, consistent with loss of H3K27me3 on the Xi. No expression changes were detected at genes that encode CTCF or hnRNPU (data not shown), which are involved in *Xist* regulation/coating [54,55]. We next examined allele-specific X-linked gene expression in Patski cells and found little evidence of reactivation from the Xi alleles after *Firre* knockdown (Figure 7B; Additional file 10: Table S5). When considering a subset of X-linked genes with ≥5 SNP-reads per 10 million mapped reads from the allele on the Xi (in control, *Firre* knockdown, or both) only 1/73 (1%) of these escape genes showed evidence of >1.5 fold upregulation, compared to other X-linked genes (23/265 or 9%) or to autosomal genes (854/11039 or 7%) (Figure 7C,D; Additional file 11: Table S6). This reflects lower levels of H3K27me3 on the Xi at escape genes (see above), resulting in a lesser impact of *Firre* knockdown compared to genes subject to XCI (Figure 6D; Additional file 7: Table S2). Taken together, our findings suggest that the lack of significant X-linked gene reactivation following depletion of *Firre* may be due to a compensatory increase in expression of genes that maintain X silencing. Such compensation reflects the multiple layers of regulation that control XCI [12,14,53].

Allele-specific expression analyses of individual genes using qRT-PCR and RT-PCR combined with restriction endonuclease digestion to selectively cleave the BL6 allele on the Xi were in general agreement with RNA-seq results. *Atp7a*, *Pgk1*, and *Iqsec2* representing genes normally subject to XCI did not show any significant expression changes after either *Firre* or *Ctcf* knockdown (Figure 7E,F). In contrast, two other genes normally subject to XCI, *Suv39h1*, and *Magee1*, were significantly upregulated, but this increased expression was from the Xa allele with no reactivation of the Xi allele (Figure 7E; Additional file 4: Table S1). Three genes that escape XCI, *Kdm5c*, *Kdm6a*, and *Eif2s3x*, showed no consistent expression changes by RT-PCR (Figure 7E; Additional file 12: Table S7). Again, *Xist* expression did not decrease but rather increased by approximately 1.5-fold after *Firre* knockdown, consistent with RNA-seq results (Figure 7E; Additional file 12: Table S7). Note that expression of *Firre* was significantly decreased (1.7-fold; *P* = 0.0008 by one-sample t-test) after *Ctcf* knockdown, suggesting that CTCF regulates expression of the lncRNA (Figure 7E).

### Gene expression analyses after *Firre* knockdown in female ES cells

To investigate the role of *Firre* in XCI initiation, we obtained a stable *Firre* knockdown using shRNA in female mouse ES cells PGK12.1 (Additional file 13: Figure S6A). Unlike what was reported for a complete *Firre* knockout, which causes a marked retardation in ES cell growth rate and colony formation [21], we did not observe dramatic growth differences between control and knockdown cells (data not shown). In addition, the master pluripotency gene *Nanog* was upregulated approximately 10-fold following differentiation, consistent with normal differentiation in knockdown PGK12.1 cells (Figure 7G). Both *Firre* knockdown and control ES cells had more than an eight-fold increase in *Xist* at day 14 compared to day 0, indicating that the knockdown did not affect *Xist* upregulation (Figure 7G). In fact, there was a 1.5- to 2-fold increase in *Xist* expression in *Firre* knockdown versus control ES cells regardless of their differentiation status, similar to what we observed in Patski cells (Additional file 13: Figure S6B). *Xist* RNA-FISH showed no changes in the shape of the *Xist* cloud between control and *Firre* knockdown cells at day 14 (Additional file 13: Figure S6C). We next used RT-PCR to measure the expression of *G6pdx*, a gene known to display a two-fold decrease in expression due to XCI upon differentiation of female ES cells [56]. *G6pdx* expression was decreased about two-fold in control and *Firre* knockdown cells at day 14 (Figure 7G), indicating that X-linked gene silencing was not disrupted after *Firre* knockdown in female ES cells. These results suggest that *Firre* does not play a major role in XCI initiation.

## Discussion

Here we have shown that the lncRNA *Firre* locus is involved in two interrelated features of the inactive X chromosome in terms of positioning near the nucleolus and maintenance of H3K27me3.

### *Firre* in nucleolar association of the Xi via CTCF

The Xi often visits the perinucleolar region during S phase in female mouse embryonic fibroblasts (MEFs) [27]. The dynamic association of the Xi to the nucleolus is cell-cycle dependent and involves the XIC, visible as a DNA-FISH signal adjacent to the edge of the nucleolus in 50% to 60% of cells, a percentage similar to what we observed for *Firre* and another lncRNA locus *Dxz4*. We postulate that the macrosatellite loci *Firre* and *Dxz4* located 54 Mb and 29 Mb away from the XIC, respectively, may provide additional attachment points to anchor the Xi via a CTCF/cohesin complex. We cannot exclude that *Firre* and *Dxz4* may also help anchor the Xi near the nuclear membrane where the Xi is also frequently found. Both the nuclear membrane and the nucleolus have been identified as preferred ‘velcro’ sites for heterochromatin [57]. The human lncRNA *FIRRE* is also bound by CTCF and RAD21 on the Xi, suggesting a conserved role between species [21,39]. Interestingly, perinucleolar targeting and H3K27me3 enrichment on the Xi are conserved in marsupials even though other mechanistic aspects of X inactivation differ and *Xist* is replaced by another lncRNA *Rsx* [58,59].

Components of the cohesin complex, RAD21 and SMC3, co-localize with CTCF at the *Firre* and *Dxz4* loci on the mouse Xi, which may help sequestration of the Xi to a specific nuclear compartment. Many but not all CTCF sites are known to attract cohesin, likely to organize chromatin within the nucleus [41-44]. Cohesin assists CTCF in targeting genomic DNA or genes to specific subnuclear locations for correct expression [60]. Furthermore, there is prior evidence that CTCF tethers chromatin to the nucleolus via a complex with nucleophosmin [33]. Thus, perinucleolar targeting of the Xi may depend on a multi-protein complex that includes CTCF, cohesin, YY1, and nucleophosmin. In addition, previous observations demonstrate that CTCF accumulation at *DXZ4* coincides with the center of the condensed Barr body in human cells, suggesting a role in Xi structure [26,37]. Recent Hi-C studies have further shown that two major domains of condensation on the human Xi are separated by the *DXZ4* region [40]. The parallel effects of *Ctcf* knockdown that we observed at *Firre* and *Dxz4* in terms of disruption of nucleolar targeting suggest that both regions may help anchor the Xi. Interestingly, CTCF helps locate another macrosatellite repeat *D4Z4* not to the perinucleolar region, but to a compartment adjacent to the nuclear membrane via a complex with A-type lamins [35]. In *Drosophila*, a protein complex containing CTCF, nucleophosmin and another nucleolar protein Modulo helps anchor centromeric chromatin to the nucleolus. Disruption of components of the complex results in abnormal spatial arrangement of the centromeric regions and in defects of heterochromatin stability and silencing [61]. Thus, while the ubiquitous protein CTCF is clearly an important factor for the correct placement of loci within the nucleus to maintain their chromatin state, this role is implemented through interactions with various other proteins (for example, cohesin, nucleophosmin, A-type lamins) that specify the subnuclear compartment. Specificity of the regions targeted to a site within the nucleus may also depend on motifs at the CTCF binding sites and on the presence of associated lncRNAs. Our re-analyses of previous data [49] are consistent with CTCF binding to *Firre* lncRNA.

The loss of CTCF binding that we observed specifically on the Xa during male and female ES cells differentiation may be mediated by the onset of DNA methylation that would preclude CTCF from binding. This is supported by our re-examination of published genome-wide profiles obtained by bisulfite sequencing that shows heavy DNA methylation at *Firre* in male mouse brain [62]. A similar mechanism has been proposed in the context of the enhancer-blocking function of CTCF at the *H19*/*Igf2* locus where DNA methylation at the imprinting control region (ICR) eliminates CTCF binding [63,64]. In human, the *DXZ4* locus is also heavily methylated on the Xa but completely unmethylated on the Xi [38,65]; however, *in vitro* DNA methylation of the CTCF binding sites apparently does not block CTCF binding, suggesting that the interactions between CTCF and DNA methylation may be more complex. Furthermore, there may be epigenetic differences between species. For example, unlike what has been reported for *DXZ4* we did not observe enrichment in H3K9me3 at the corresponding mouse locus [38] (data not shown). Furthermore, we did not detect overlap of the *Dxz4* and *Firre* loci, which differs from a previous study indicating aggregation of *FIRRE* and *DXZ4* via CTCF in human cells [39,40].

### *Firre* in maintenance of H3K27me3 on the Xi

Studies in yeast have proposed that lncRNAs may function as assembly platforms for the recruitment of proteins such as histone methyltransferases to heterochromatin nucleation sites [66]. Our findings support a role for the *Firre* lncRNA in preserving one of the epigenetic characteristics associated with XCI, probably via specific proteins that control H3K27me3. Indeed, expression of *Suz12*, a gene that encodes a core component of the PRC2 complex for the deposition of H3K27me3 on the Xi was decreased after *Firre* RNA knockdown. Additional studies will be required to fully understand the molecular mechanisms that govern epigenetic features of the Xi in relation to nuclear elements such as the nucleolus or the nuclear membrane. A similar type of anchoring mechanism for the regulation of a specific epigenetic feature has been proposed for the lncRNA *Kcnq1ot1* that contacts the nucleolus possibly via CTCF and controls H3K27me3 levels at the imprinted locus *Kcnq1* [67]. However, perinucleolar localization of *Kcnq1ot1* may not be sufficient to preclude transcription of genes in the imprinted domain [68]. Similarly, we did not observe reactivation of X-linked genes upon *Firre* knockdown, consistent with H3K27me3 representing only one layer of XCI control. A new study supports a role for *Firre* in nuclear organization via interactions with the nuclear matrix factor hnRNPU to help co-localize genomic loci involved in adipogenesis by *cis*- and *trans*-interactions [21]. Interestingly, hnRNPU is required for *Xist* association with the Xi and deposition of H3K27me3 [55].

A similar number of autosomal and X-linked genes were upregulated or downregulated, after *Firre* knockdown in fibroblasts probably due to indirect effects of disrupting X heterochromatin and to loss of H3K27me3. Importantly, our results are consistent with *Firre* having a role in maintenance of H3K27me3 independent of *Xist* that was not decreased by *Firre* knockdown. The lack of X-linked gene reactivation we observed following knockdown of either *Firre* or *Ctcf* is not surprising given that silencing of the Xi is extremely stable in somatic cells even after depletion of *Xist* or of PRC2 [9,15,69,70]. Even removal of DNA methylation by 5-aza-cytidine does not result in substantial X-linked gene reactivation in somatic cells [71]. Thus, *Xist* coating, DNA methylation, and histone modifications all contribute in synergism to maintain X silencing [11]. Interestingly, among those with increased expression upon *Firre* knockdown were genes that encode components of the PRC1 complex (*Bmi1*, *Cbx6*, and *Rnf2*) and other proteins that also control heterochromatin (*Suv39h1*, *Cbx1*) and deposit silencing marks on the Xi [6,13], as well as *Dnmt1* implicated in maintenance of DNA methylation, suggesting compensatory silencing mechanisms in response to the loss of H3K27me3. Our analyses were done using short-term knockdown and would not detect long-term effects on gene expression. It is possible that failure to maintain perinucleolar anchoring of the Xi would eventually affect gene expression after multiple cell divisions. Such a delayed effect was demonstrated in the case of *Xist* whose continued expression appeared dispensable for maintenance of XCI in short-term analyses of cell lines [9], while being essential for long-term maintenance of XCI [10]. Our findings of dramatic differences in CTCF binding between males and females in differentiated ES cells and adult tissues but not in undifferentiated ES cells are consistent with *Firre* regulating maintenance rather than initiation of XCI. Furthermore, *Firre* knockdown did not affect XCI onset in female ES cells.

## Conclusions

In summary, we show that *Firre*, a lncRNA transcribed from a gene that escapes XCI contributes to the maintenance of a silenced X chromosome compartment by stabilizing one of the main repressive histone marks on the Xi. Specific CTCF/cohesin binding to the *Firre* locus on the Xi and/or the lncRNA itself may play a role in positioning the Xi near the nucleolus surface and in maintaining one of its epigenetic features. Our results are consistent with XCI being insured by the synergy of multiple layers of control.

## Methods

### Mouse embryos, tissues, and cell lines

Embryos collected from pregnant C57BL/6J (BL6) mice at 12.5 dpc were sexed using PCR primers for the male-specific gene, *Sry*. Male and female BL6 adult livers were collected. The Patski fibroblast line in which the Xi is from BL6 and the Xa from *M. spretus* was originally derived from embryonic kidney [17,46]. Female ES cells (PGK12.1) [72], and male ES cells (WD44) were cultured in standard ES medium with 1,000 U/mL leukemia inhibitory factor (LIF) (Millipore) on MEF feeders, and harvested or replated for other experiments after depletion of MEF feeders. Mouse ES cells were differentiated using the standard embryoid body (EB) protocol. The presence of normal X chromosomes was verified by karyotyping. Brains were collected from female F1 obtained by mating *M. spretus* males (Jackson Labs) with females that carry an *Xist* mutation (B6.Cg-Xist < tm5Sado>) [73]. F1 mice carrying the mutant *Xist* allele fail to silence the BL6 X and thus have completed skewing of inactivation of the *spretus* X. Primary fibroblasts cultures were derived from ears of male and female BL6 mice. Primary neuron cultures were established from the hippocampus dissected from 0 to 2-day-old mouse pups.

### ChIP-chip

Chromatin immunoprecipitations [74] were performed using 10 μg of CTCF antibody (Millipore), 10 ug of RAD21 antibody (Abcam), or 5 μg of H3K27me3 antibody (Millipore) as previously described [17]. ChIP-chip was done using Nimblegen genome 385 K and 2.1 M tiling arrays to cover the entire X (100 bp tiling interval). H3K27me3 ChIP-chip before and after transient knockdown of either *Firre* or *Ctcf* in Patski cells were done using Nimblegen 2.1 M promoter arrays (100 bp interval). H3K27me3 ChIP-chip before and after stable knockdown of *Firre* (siRNA and shRNA) in Patski cells were done using Agilent 1 M mouse promoter arrays (200 bp interval). GFF ratios (log_2_ ChIP/input) and peak files were generated using Nimblescan or Agilent DNA Feature extraction software. Peaks were identified using a 500 bp sliding window with default values from Nimblescan. Metagene profiles were obtained by calculating averages of H3K27me3 enrichment at the 5′ end of genes using a 500 bp sliding window (100 bp interval) for NimbleGen arrays, and a 600 bp sliding window (200 bp interval) for Agilent arrays by end-analysis [75]. Only unique RefSeq genes were included in the analysis. H3K27me3 enrichment at the gene body (1 to 2 kb downstream of TSS) of approximately 600 X-linked and approximately 16,000 autosomal genes was compared for each gene to generate scatter plots (Figure 6E, F). Verification of CTCF binding sites by gel mobility shift assays was done as described [76].

### Allele-specific ChIP-seq

Allele-specific ChIP-seq was done as described [47]. Four or five CTCF ChIP samples were pooled (approximately 200 to 300 ng) for library preparation. The library with inserts of 200 to 600 bp was sequenced on Illumina Genome Analyzer, yielding 36 nt single-end reads for ChIP-seq in Patski cells or 100 nt pair-end reads for ChIP-seq in brain. A total of 49.3 M and 283.7 M uniquely mapped reads were obtained for Patski cells and brain, respectively. A pseudo-*spretus* genome was assembled by substituting available SNPs between BL6 and *M. spretus* into the BL6 reference genome (mm9). SNPs were obtained from the Sanger Institute (SNP database Nov/2011 version) and from our previous study (169,031 additional SNPs, of which 3,571 were X-linked) [17]. Reads were aligned separately to the BL6 reference genome and to the pseudo-*spretus* genome using BWA/v0.5.9 with default parameters. Reads that mapped uniquely and with high-quality mapping score (MAPQ ≥30) to either the BL6 genome or the pseudo-*spretus* genome were segregated into three categories: (1) BL6-SNP reads containing only BL6-specific SNPs; (2) *spretus*-SNP reads containing only *spretus*-specific SNPs; (3) ambiguous reads that do not contain SNPs or contain both BL6-specific SNPs and *spretus*-specific SNPs. The number of total mapped reads and allele-specific reads in each 100 bp window was calculated and viewed in the UCSC genome browser (Figure 2A). For PolII-S5p ChIP-seq the same allelic analysis was done using 100 nt single-end reads obtained by Hi-seq in Patski cells. In addition, we re-analyzed PolII-S5p ChIP Hi-seq datasets we previously obtained in mouse brain (GSE30761) [47]. PolII-S5p allelic profiles were viewed in the UCSC genome browser (Figure 2B).

### Immunofluorescence, RNA-FISH, and DNA-FISH

Cells cultured on chamber slides or, in the case of neuronal cells, on poly-Lysine pre-coated coverslips for 6-day cultures were permeabilized in 0.5% triton X-100 for 10 min and fixed with 4% paraformaldehyde for 10 min. Fixed cells were used for RNA-FISH or DNA-FISH directly using standard protocols. For immunofluorescence (IF), fixed cells were blocked in BSA buffer (4XSSC, 0.42% BSA) for 30 min. After incubation with a primary antibody for nucleophosmin (Abcam) or for H3K27me3 (Millipore) at 1:500 dilution overnight at 4°C cells were washed with PBST and incubated with the secondary antibody conjugated to Texas Red or AMCA (Vectashield) at 37°C for 1 h. DNA-FISH was done using BAC probes (RP23-338M16 or RP24-322N20 for *Firre* and RP23-299L1 for *Dxz4*), and RNA-FISH using an *Xist* plasmid or *Firre* BAC (RP24-322N20). Probes were labeled with SpectrumGreen or SpectrumRed dUTP (Vysis). For IF combined with RNA-FISH, cells after IF were hybridized with probes overnight at 37°C and washed in 50% formamide/2×SSC and 2×SSC at 42°C, prior to Hoechst 33342 (Molecular probes) counterstaining. For IF combined with DNA-FISH, cells after IF were post-fixed in 4% paraformaldehyde for 10 min, denatured in 70% formamide/2×SSC at 85°C for 15 min, dehydrated for 2 min each in 70%, 85%, and 100% ethanol, prior to overnight hybridization at 42°C and washing as described [77]. The association frequency between *Firre* (or *Dxz4*) and the nucleolus was scored using a Deltavision deconvolution microscope (Keck Center, University of Washington). At least 100 nuclei were examined in each condition.

### *Ctcf* and *Firre* RNAi knockdown

A pool of two ‘Silencer select’ siRNA duplexes for *Firre* knockdown (duplex1: sense, 5′-CAGGUACCGUGAGCAAUCAtt-3′, antisense, 5′-UGAUUGCUCACGGUACCUGgt-3′, duplex2: sense, 5′-CCUUCAGAGUAUUAAUGCAtt-3′, antisense, 5′-UGCAUUAAUACUCUGAAGGgt-3′) and a ‘Stealth’ 25-mer siRNA duplex for *Ctcf* knockdown (sense, 5′-UGGACCAGCACAGUUAUCUGCAUGU-3′, antisense, 5′-ACAUGCAGAUAACUGUGCUGGUCCA-3′) were obtained with a control Stealth oligomer with no specificity to the mouse genome (Invitrogen). Double siRNA transfections were performed using 5 μL of lipofectamine mixed with 250 μL of Opti-MEM® I Reduced Serum Medium containing 100 pmol of each RNAi oligomer. After 20 min incubation the mix was added to 2 × 10^5^ cells in 1.25 mL DMEM/5%FBS with no antibiotics. The medium was changed with DMEM/10% FBS after incubation at 37°C for 16 to 24 h. Two days after the first transfection, cells were split and 2 × 10^5^ cells transfected again. Three days later cells were harvested and immunostaining, western blots, and qRT-PCR performed to verify knockdown efficiency. Stable knockdown using *Firre* shRNA that hit five regions exclusively in the *Firre* gene (Additional file 1: Figure S1) was done using Addgene’s pLKO.1 protocol [78]. Forward oligo, 5′ CCGG-CCTTCAGAGTATTAATGCAGA (sense)-CTCGAG-TCTGCATTAATACTCTGAAGG (antisense)-TTTTTG 3′, and reverse oligo, 5′ AATTCAAAAA-CCTTCAGAGTATTAATGCAGA (sense)-CTCGAG-TCTGCATTAATACTCTGAAGG (antisense) 3′ (IDT) were annealed for cloning into the pLKO.1 vector, as well as control oligos with scramble sequence (Addgene) to generate the control vector. The two plasmids were then used to produce lentiviral particles (Fred Hutchinson Cancer Research Center/CCEH shared resources) for infection of Patski cells and puromycin selection for cells stably expressing shRNA. The knockdown efficiency was determined by qRT-PCR. In the experiments to confirm the effect of *Firre* depletion of H3K27me3 by ChIP-chip and to examine gene expression changes, control and stable knockdown Patski cells were subject to an additional transfection with control and *Firre* siRNA, respectively, to achieve maximum depletion. Mouse female ES cells (PGK12.1) with control and stable *Firre* knockdown were established using the same lentiviral particles.

### RNA-seq

RNA-seq indexed libraries were prepared using Illumina TruSeq RNA sample preparation kit for two biological replicates from control or stable knockdown (si&shRNA) Patski cells, respectively. Libraries from two replicates were pooled for one lane of 36 nt single-end plus index sequencing as described [17]. Reads were mapped and RPKM obtained using TopHat/Cufflinks [79]. A total of 22.3 M and 23.7 M mapped reads were obtained from two controls (11.8 M and 10.5 M) and two *Firre* stable knockdown (11.9 M and 11.7 M) replicates, respectively. Differential gene expression was determined by Cuffdiff2 [80]. Functional analysis of upregulated and downregulated genes was done by GO DAVID [52]. Allele-specific RNA-seq analysis was performed as described above. Reads were aligned separately to the BL6 reference genome and transcriptome (mm9) and to the pseudo-*spretus* genome and transcriptome using Tophat/v2.0.0 with default parameters. Only those reads that mapped uniquely and with a high-quality mapping score (MAPQ ≥30) to either the BL6 genome or the pseudo-*spretus* genome were kept for allele-specific analyses. SNP-read counts from exons of BL6 and *spretus* alleles were calculated and adjusted by the ratio of the total number of mapped reads between control and knockdown (that is, the SNP-read counts from the knockdown were divided by 1.06 (23.7 M/22.3 M). Since *Eif2s3x* exons (except exon 1) have a high sequence similarity to another X region (chrX:31680780-31684279), we included reads contained in exons with a low MAPQ score for this gene. For *Firre* we included SNP reads from both exonic and intronic regions.

### Quantitative RT-PCR and allele-specific restriction enzyme digestion

RNA extracted from either mouse tissues or cells by the Qiagen RNeasy kit with on-column DNaseI digestion was reverse transcribed into first-strand cDNAs using SuperScriptII reverse transcriptase (Invitrogen). qRT-PCR was done using gene-specific primers and an ABI7900 PCR system. The primer sequence information is presented in Additional file 14: Table S8. The primers used for *Firre* qRT-PCR spanning an intron produce an amplicon targeting most of *Firre* transcripts (Additional file 1: Figure S1). To measure allele-specific expression cDNA was used for 32-cycle PCR with primers designed around the SNPs. PCR products were split into two aliquots, one serving as undigested control while the other was digested with a restriction endonuclease (SnabI for *Atp7a*, BglII for *Pgk1*, and KpnI for *Iqsec2*) (New England Biolabs). Gel electrophoresis was performed to examine whether reactivation happened after *Firre* or *Ctcf* knockdown.

### Statistical analyses

Two-tailed unpaired student t-tests were used to compare the frequency of association of *Firre* and *Dxz4* to the nucleolus surface between females and males, and between *Firre* or *Ctcf* knockdown and control Patski cells. Gene expression ratios between *Firre* or *Ctcf* knockdown cells and control cells were compared to a ratio of 1 expected if there was no change in expression using one-sample t-tests. Chi-square tests were done to evaluate changes in the frequency of intense H3K27me3 staining spots between *Firre* or *Ctcf* knockdown and control Patski cells. Kolmogorov-Smirnov-tests were used to determine the significance of changes in the distribution of H3K27me3 enrichment within 1 to 2 kb downstream of the TSS of X-linked or autosomal genes (Figure 6C). Significances of gene expression fold changes measured by RNA-seq analysis of two control and two *Firre* stable knockdown Patski cells were determined by Cuffdiff2 [80].

### Ethics statement

For mice sacrificed, euthanasia was accomplished using two methods (carbon dioxide asphyxiation followed by cervical dislocation) as required by the University of Washington’s Office of Animal Welfare. Husbandry and all other procedures were approved by the University of Washington’s Office of Animal Welfare (Protocol 2254).

## Additional files

Additional file 1: Figure S1. Location of qRT-PCR amplicon and of shRNA hits in the *Firre* gene viewed in the UCSC genome browser. The *Firre* qRT-PCR amplicon spans an intron and targets most transcripts. The shRNAs hit five regions in *Firre*, two in exons and three in introns. There is no other hit in the mouse genome from Blat search. Additional file 2: Figure S2. RNA-FISH fails to detect a *Firre* signal on the Xi in Patski cells and detects no changes in *Xist* cloud after *Firre* knockdown. **(A)** Examples of RNA-FISH for *Firre* (red) and *Xist* (green) to mark the Xi in nuclei of Patski cells. The single bright *Firre* signal that does not overlap with *Xist* presumably corresponds to the Xa locus (arrow). **(B)** No change in the size or shape of *Xist* RNA clouds is detected in Patski cells after *Firre* knockdown. Examples of *Xist* (green) RNA FISH in nuclei of Patski cells after *Firre* knockdown using shRNA. Additional file 3: Figure S3. RNA FISH fails to detect *Firre* transcripts on the Xi in female MEFs and primary neurons. **(A)** Examples of MEF nuclei subject to RNA-FISH to detect *Xist* (red) and *Firre* (green). Note that one or more bright *Firre* signals are detected but none overlap with the two *Xist* clouds that mark the two Xi in this MEF line. **(B)** Same analysis in primary neurons in which only one Xi is present. Additional file 4: Table S1. Genes with significant expression changes between control and *Firre* knockdown Patski cells. Additional file 5: Figure S4. Examples of nuclei from fibroblasts that demonstrate the presence of association (A, D) or absence of association (B, C, E) between *Firre* and *Dxz4* (A, B, C), or *Dxz4* and *Xist* (D, E), with the nucleolus (see also Figure 4). **(A, B)** Nuclei from female ear fibroblasts subjected to DNA-FISH to detect *Firre* (red), *Dxz4* (green), and immunostaining to detect nucleophosmin (blue). **(C)** Same analysis in male ear fibroblasts. **(D, E)** Nuclei from female Patski cells subjected to DNA-FISH to detect *Dxz4* (red), RNA-FISH to detect *Xist* (green) and mark the Xi, and immunostaining to detect nucleophosmin (blue). One deconvoluted Z section is shown. Additional file 6: Figure S5. Efficiency of *Ctcf* knockdown and H3K27me3 changes after *Firre* stable knockdown. **(A)** qRT-PCR (left) and western blots (right) confirm *Ctcf* knockdown in Patski cells using siRNA. Error bars indicate s.e.m. **(B)** H3K27me3 enrichment is reduced at the 5′ end of X-linked genes (X) but not autosomal genes (A) after *Firre* stable knockdown in Patski cells using shRNA and siRNA. Metagene analysis shows average H3K27me3 enrichment (log2 ChIP/input) 3 kb upstream and downstream of the transcription start site (TSS) for 647 X-linked versus 16,141 autosomal genes. See also Figure 6B. Note the reduction in H3K27me3 at the TSS in Figure 6B is probably due to artifacts from hybridization or types of promoter array used, which is not observed here. Additional file 7: Table S2. H3K27me3 enrichment at 10 escape genes in control and *Firre* knockdown Patski cells. Additional file 8: Table S3. Functional analysis of genes significantly misregulated upon *Firre* knockdown in Patski cells. Additional file 9: Table S4. Expression changes for genes involved in heterochromatin and gene silencing between *Firre* knockdown and control Patski cells. Additional file 10: Table S5. Number of alleles with expression changes between control and *Firre* knockdown Patski cells. Additional file 11: Table S6. Expression changes for genes with ≥5 SRPM on the Xi after *Firre* knockdown in Patski cells. Additional file 12: Table S7. Expression changes for escape genes after *Firre* knockdown in Patski cells. Additional file 13: Figure S6.
*Firre* stable knockdown in female mouse ES cells did not disrupt *Xist* upregulation/coating during differentiation. **(A)** Evidence of stable *Firre* knockdown (KD) in female mouse ES cells PGK12.1 using shRNA. qRT-PCR was performed at day 3 and day 10 after infection with lentiviral particles carrying control scramble shRNA or *Firre* shRNA. Puromycin was added 1 day after infection to select cells expressing shRNA. **(B)** Similar *Xist* expression fold change are observed in undifferentiated and differentiated PGK12.1 ES cells (see also Figure 7G) and in Patski cells (see also Figure 7E) upon *Firre* knockdown. **(C)** *Xist* cloud shape and size is similar between control and knockdown PGK12.1 cells at day14. Examples of *Xist* (green) RNA-FISH in three nuclei for each condition are shown. Additional file 14: Table S8. Sequences of primers.
