# Supplementary material for: The lncRNA Firre anchors the inactive X chromosome to the nucleolus by binding CTCF and maintains H3K27me3 methylation
Source: Genome Biol. 2015 Mar 12;16(1):52. doi: 10.1186/s13059-015-0618-0 (PMC4391730; doi:10.1186/s13059-015-0618-0)
Supplement: Additional file 1: Figure S1. — Location of qRT-PCR amplicon and of shRNA hits in the Firre gene viewed in the UCSC genome browser. The Firre qRT-PCR amplicon spans an intron and targets most transcripts. The shRNAs hit five regions in Firre, two in exons and three in introns. There is no other hit in the mouse genome from Blat search. [file 13059_2015_618_MOESM1_ESM.pdf]

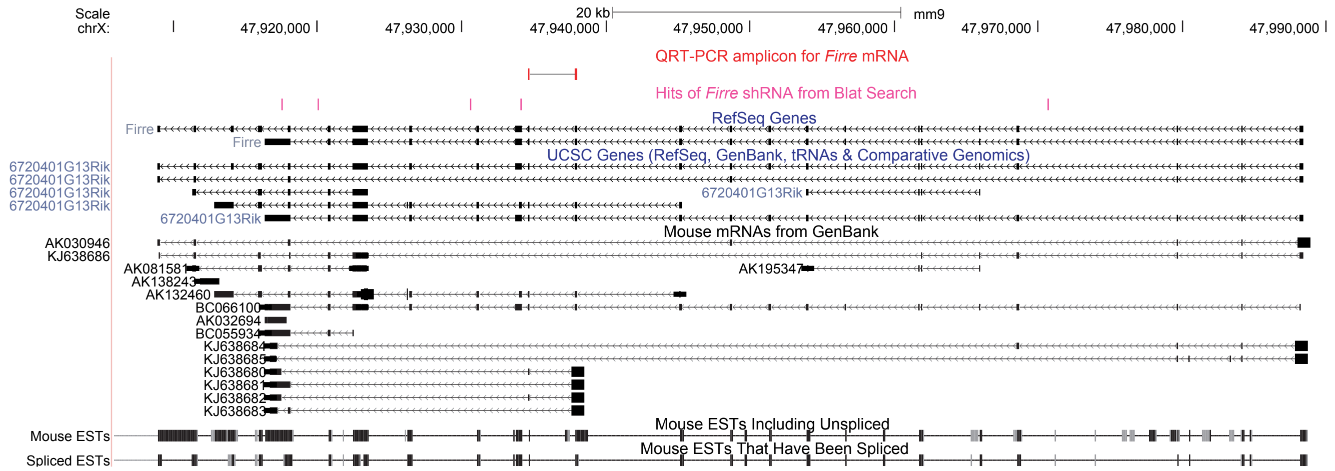

Figure S1. Location of qRT-PCR amplicon and of shRNA hits in the *Firre* gene viewed in the UCSC genome browser. The *Firre* qRT-PCR amplicon spans an intron and targets most transcripts. The shRNAs hit five regions in *Firre*, two in exons and three in introns. There is no other hit in the mouse genome from Blat search.
