# Supplementary material for: The lncRNA Firre anchors the inactive X chromosome to the nucleolus by binding CTCF and maintains H3K27me3 methylation
Source: Genome Biol. 2015 Mar 12;16(1):52. doi: 10.1186/s13059-015-0618-0 (PMC4391730; doi:10.1186/s13059-015-0618-0)
Supplement: Additional file 2: Figure S2. — RNA-FISH fails to detect a Firre signal on the Xi in Patski cells and detects no changes in Xist cloud after Firre knockdown. (A) Examples of RNA-FISH for Firre (red) and Xist (green) to mark the Xi in nuclei of Patski cells. The single bright Firre signal that does not overlap with Xist presumably corresponds to the Xa locus (arrow). (B) No change in the size or shape of Xist RNA clouds is detected in Patski cells after Firre knockdown. Examples of Xist (green) RNA FISH in nuclei of Patski cells after Firre knockdown using shRNA. [file 13059_2015_618_MOESM2_ESM.pdf]

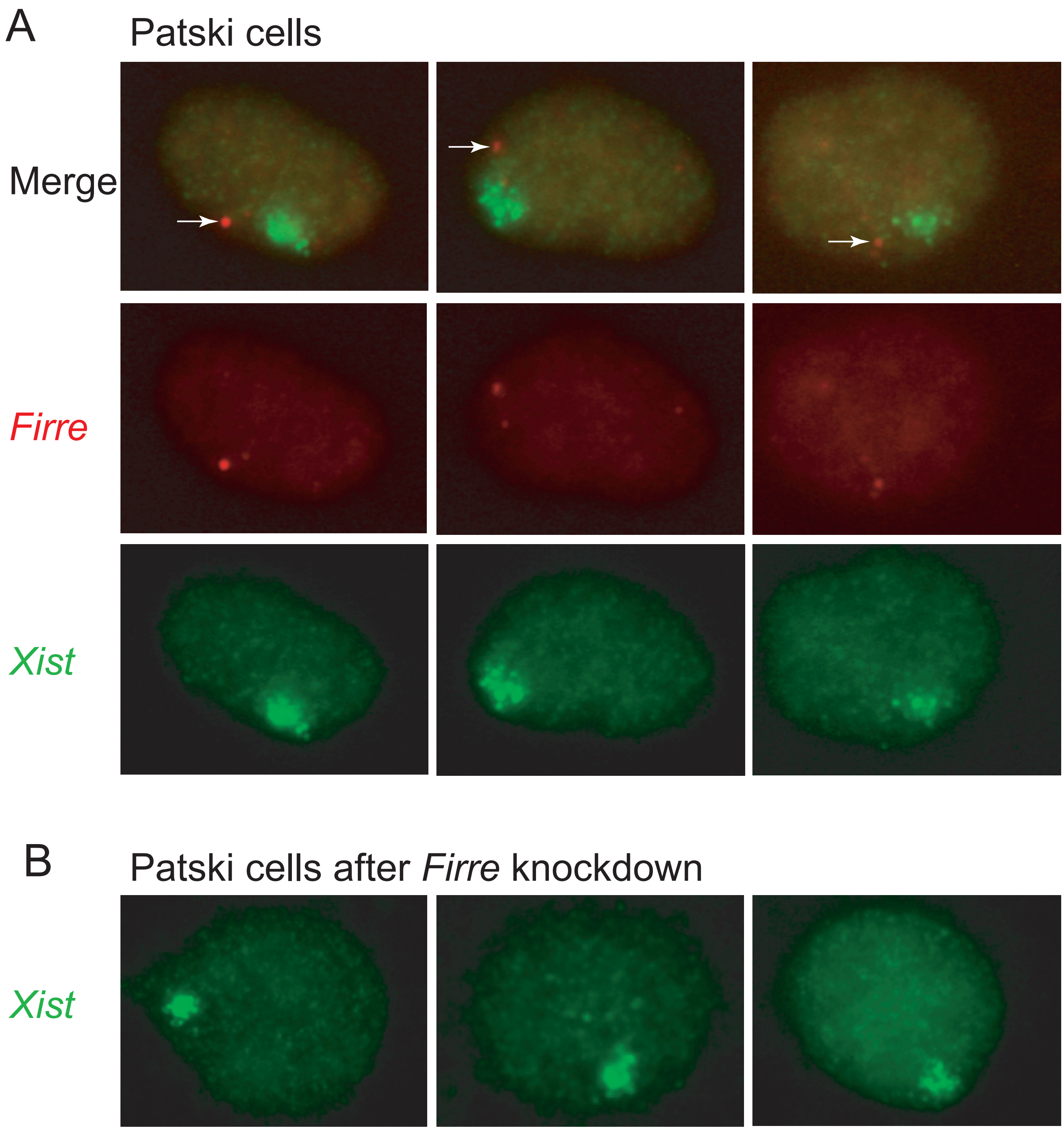

Figure S2. RNA-FISH fails to detect a *Firre* signal on the Xi in Patski cells and detects no changes in *Xist* cloud after *Firre* knockdown. **(A)** Examples of RNA-FISH for *Firre* (red) and *Xist* (green) to mark the Xi in nuclei of Patski cells. The single bright *Firre* signal that does not overlap with *Xist* presumably corresponds to the Xa (arrow). **(B)** No change in the size or shape of *Xist* RNA clouds was detected in Patski cells after *Firre* knockdown. Examples of *Xist* (green) RNA FISH in nuclei of Patski cells after *Firre* knockdown using shRNA.
