# Supplementary material for: The lncRNA Firre anchors the inactive X chromosome to the nucleolus by binding CTCF and maintains H3K27me3 methylation
Source: Genome Biol. 2015 Mar 12;16(1):52. doi: 10.1186/s13059-015-0618-0 (PMC4391730; doi:10.1186/s13059-015-0618-0)
Supplement: Additional file 3: Figure S3. — RNA FISH fails to detect Firre transcripts on the Xi in female MEFs and primary neurons. (A) Examples of MEF nuclei subject to RNA-FISH to detect Xist (red) and Firre (green). Note that one or more bright Firre signals are detected but none overlap with the two Xist clouds that mark the two Xi in this MEF line. (B) Same analysis in primary neurons in which only one Xi is present. [file 13059_2015_618_MOESM3_ESM.pdf]

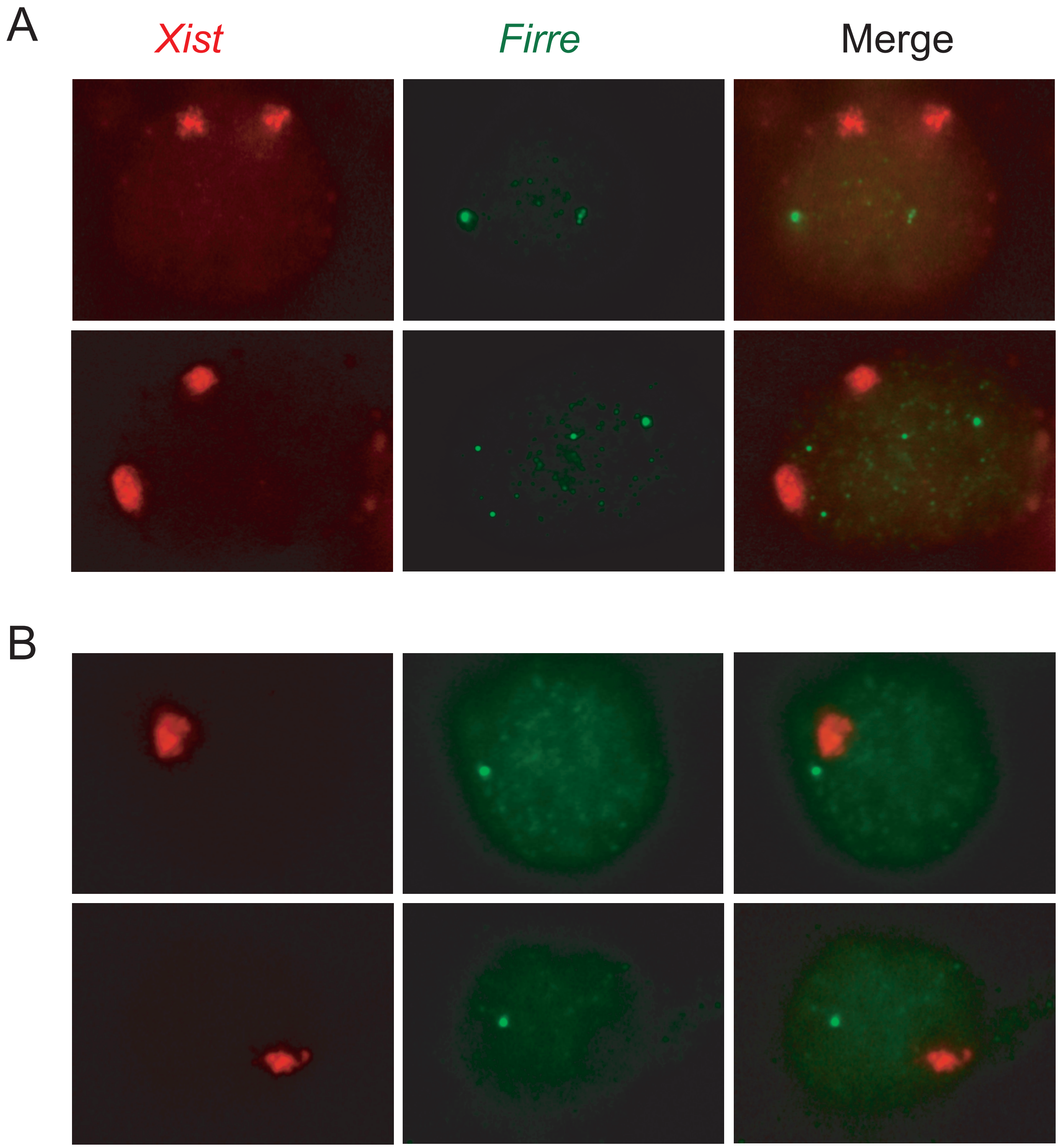

Figure S3 . RNA FISH fails to detect *Firre* transcripts on the Xi in female MEFs (A) and primary neurons (B). **(A)** Examples of nuclei subject to RNA-FISH to detect *Xist* (red) and mark the Xi, and to detect *Firre* (green). Note that one or more bright *Firre* signals were detected but none overlapped with the two *Xist* clouds, which marked the two Xi in this MEF line. **(B)** Same analysis in primary neurons in which only one Xi is present.
