# Supplementary material for: The lncRNA Firre anchors the inactive X chromosome to the nucleolus by binding CTCF and maintains H3K27me3 methylation
Source: Genome Biol. 2015 Mar 12;16(1):52. doi: 10.1186/s13059-015-0618-0 (PMC4391730; doi:10.1186/s13059-015-0618-0)
Supplement: Additional file 5: Figure S4. — Examples of nuclei from fibroblasts that demonstrate the presence of association (A, D) or absence of association (B, C, E) between Firre and Dxz4 (A, B, C), or Dxz4 and Xist (D, E), with the nucleolus (see also Figure 4). (A, B) Nuclei from female ear fibroblasts subjected to DNA-FISH to detect Firre (red), Dxz4 (green), and immunostaining to detect nucleophosmin (blue). (C) Same analysis in male ear fibroblasts. (D, E) Nuclei from female Patski cells subjected to DNA-FISH to detect Dxz4 (red), RNA-FISH to detect Xist (green) and mark the Xi, and immunostaining to detect nucleophosmin (blue). One deconvoluted Z section is shown. [file 13059_2015_618_MOESM5_ESM.pdf]

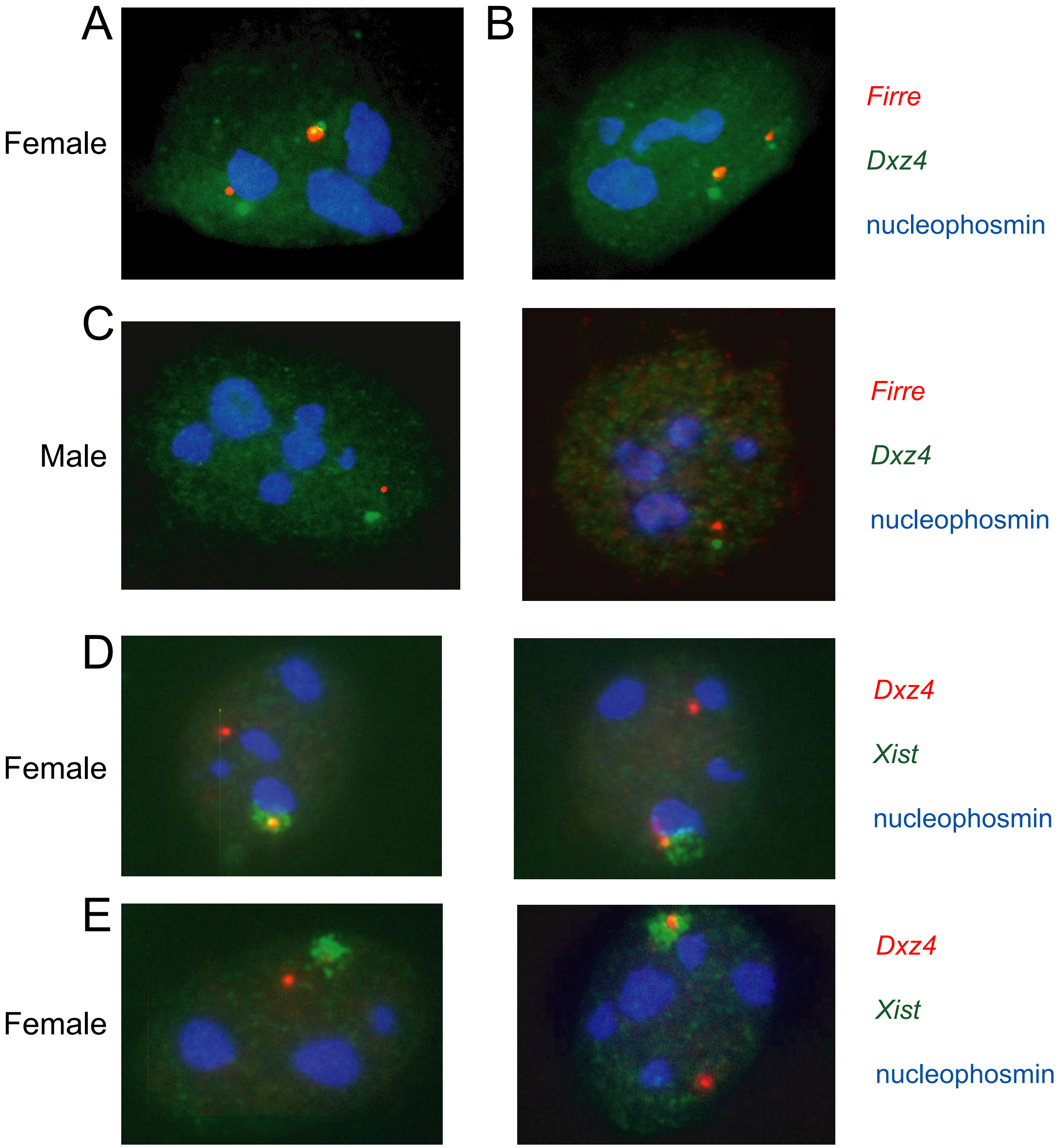


**Figure S4.** Examples of nuclei from fibroblasts with (A, D) and without (B, C, E) association of *Firre* and *Dxz4* (A, B, C), or *Dxz4* and *Xist* (D, E), with the nucleolus (see also Figure 4).
**(A, B)** Nuclei from female ear fibroblasts (A, B) subjected to DNA-FISH to detect *Firre* (red), *Dxz4* (green), and immunostaining to detect nucleophosmin (blue).
**(C)** Same analysis in male ear fibroblasts.
**(D, E)** Nuclei from female Patski cells subjected to DNA-FISH to detect *Dxz4* (red) and to RNA-FISH to detect *Xist* (green) and mark the Xi, and immunostaining to detect nucleophosmin (blue).
One de-convoluted Z section is shown.
