# Supplementary material for: The lncRNA Firre anchors the inactive X chromosome to the nucleolus by binding CTCF and maintains H3K27me3 methylation
Source: Genome Biol. 2015 Mar 12;16(1):52. doi: 10.1186/s13059-015-0618-0 (PMC4391730; doi:10.1186/s13059-015-0618-0)
Supplement: Additional file 6: Figure S5. — Efficiency of Ctcf knockdown and H3K27me3 changes after Firre stable knockdown. (A) qRT-PCR (left) and western blots (right) confirm Ctcf knockdown in Patski cells using siRNA. Error bars indicate s.e.m. (B) H3K27me3 enrichment is reduced at the 5′ end of X-linked genes (X) but not autosomal genes (A) after Firre stable knockdown in Patski cells using shRNA and siRNA. Metagene analysis shows average H3K27me3 enrichment (log2 ChIP/input) 3 kb upstream and downstream of the transcription start site (TSS) for 647 X-linked versus 16,141 autosomal genes. See also Figure 6B. Note the reduction in H3K27me3 at the TSS in Figure 6B is probably due to artifacts from hybridization or types of promoter array used, which is not observed here. [file 13059_2015_618_MOESM6_ESM.pdf]

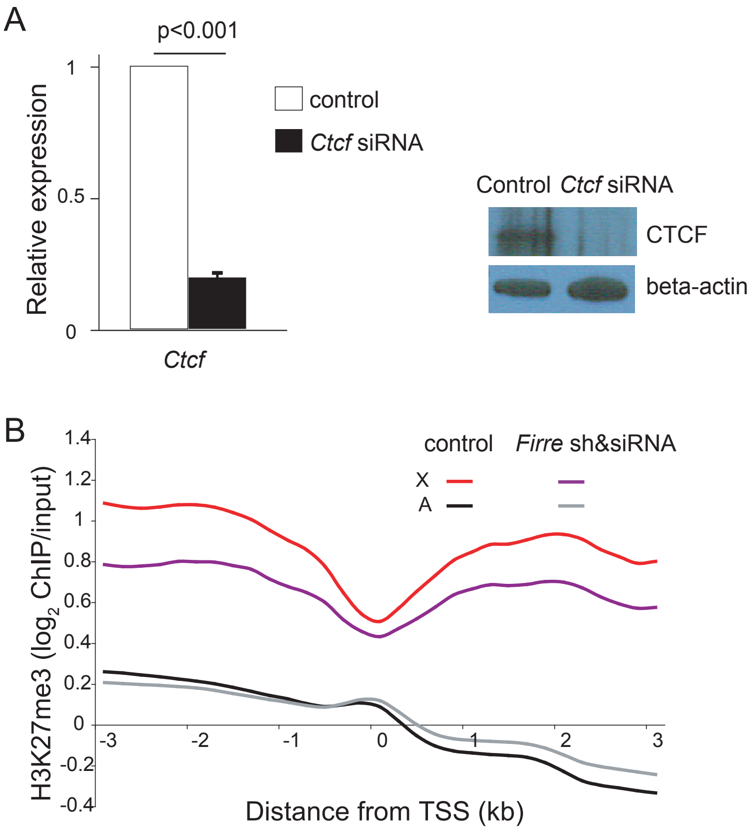

Figure S5. Efficiency of *Ctcf* knockdown and H3K27me3 changes after *Firre* stable knockdown. **(A)** qRT-PCR (left) and Western blots (right) confirm *Ctcf* knockdown in Patski cells using siRNA. Error bars indicate s.e.m. **(B)** H3K27me3 enrichment is reduced at the 5' end of X-linked genes (X) but not autosomal genes (A) after *Firre* stable knockdown in Patski cells using shRNA and siRNA. Metagene analysis shows average H3K27me3 enrichment (log2 ChIP/input) 3kb upstream and downstream of the transcription start site (TSS) for 647 X-linked versus 16141 autosomal genes. See also Figure 6B. Note the reduction in H3K27me3 at the TSS in Figure 6B is probably due to artifacts from hybridization or types of promoter array used, which is not observed here.
