# Supplementary material for: The lncRNA Firre anchors the inactive X chromosome to the nucleolus by binding CTCF and maintains H3K27me3 methylation
Source: Genome Biol. 2015 Mar 12;16(1):52. doi: 10.1186/s13059-015-0618-0 (PMC4391730; doi:10.1186/s13059-015-0618-0)
Supplement: Additional file 13: Figure S6. — Firre stable knockdown in female mouse ES cells did not disrupt Xist upregulation/coating during differentiation. (A) Evidence of stable Firre knockdown (KD) in female mouse ES cells PGK12.1 using shRNA. qRT-PCR was performed at day 3 and day 10 after infection with lentiviral particles carrying control scramble shRNA or Firre shRNA. Puromycin was added 1 day after infection to select cells expressing shRNA. (B) Similar Xist expression fold change are observed in undifferentiated and differentiated PGK12.1 ES cells (see also Figure 7G) and in Patski cells (see also Figure 7E) upon Firre knockdown. (C) Xist cloud shape and size is similar between control and knockdown PGK12.1 cells at day14. Examples of Xist (green) RNA-FISH in three nuclei for each condition are shown. [file 13059_2015_618_MOESM13_ESM.pdf]

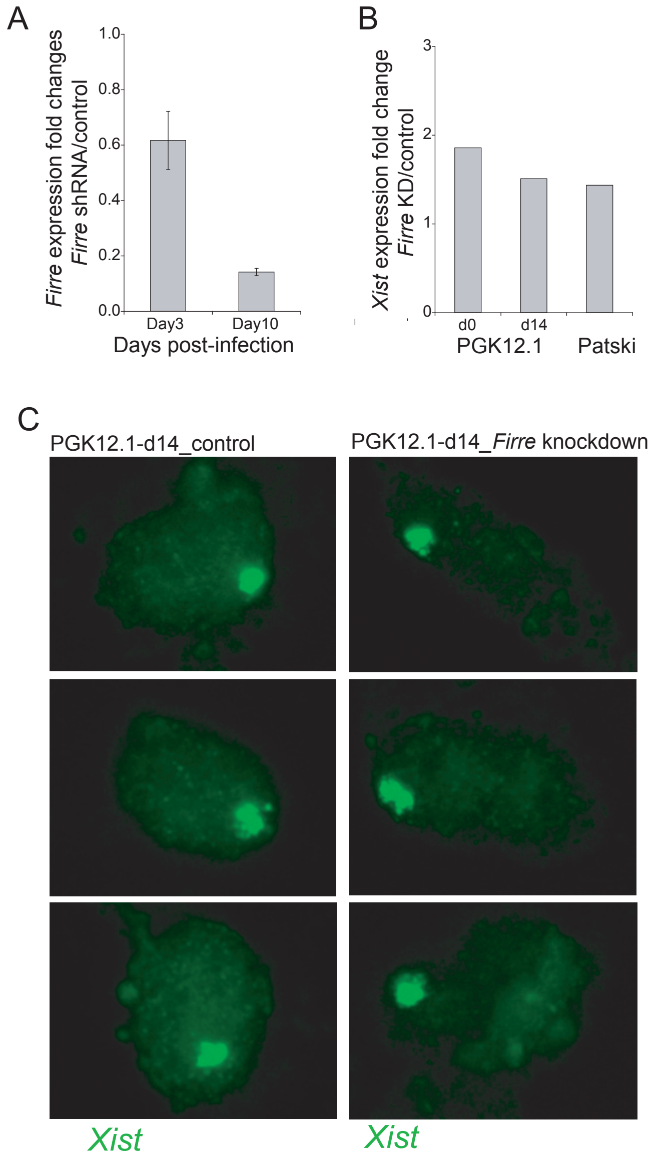

Figure S6. *Firre* stable knockdown in female mouse ES cells did not disrupt *Xist* upregulation/coating during differentiation. **(A)** Evidence of stable *Firre* knockdown (KD) in female mouse ES cells PGK12.1 using shRNA. qRT-PCR was performed at day 3 and day 10 after infection with lentiviral particles carrying control scramble shRNA or *Firre* shRNA. Puromycin was added one day after infection to select cells expressing shRNA. **(B)** Similar *Xist* expression fold change are observed in undifferentiated and differentiated PGK12.1 ES cells (see also Fig. 7G) and Patski cells (see also Fig. 7E) upon *Firre* knockdown. (C) *Xist* cloud shape and size is similar between control and knockdown PGK12.1 cells at day14. Examples of *Xist* (green) RNA FISH in three nuclei for each condition are shown.
